# Ultrasound-Guided Rhomboid Block versus Paravertebral Block in Postoperative Analgesia for Video-Assisted Thoracoscopic Surgery: A Prospective Randomized Controlled Clinical Trial

**DOI:** 10.1155/2023/3924511

**Published:** 2023-03-01

**Authors:** Yan Wang, Xiaoping Gu, Simin Huang, Minke Shi, Xiaofeng He, Zhengliang Ma

**Affiliations:** ^1^Department of Anesthesiology, Nanjing Drum Tower Hospital Clinical College of Nanjing Medical University, Nanjing, China; ^2^Nanjing Drum Tower Hospital Clinical College of Nanjing Medical University, Nanjing, China; ^3^Department of Thoracic Surgery, Nanjing Drum Tower Hospital Clinical College of Nanjing Medical University, Nanjing, China

## Abstract

**Introduction:**

The anesthetic efficacy of the ultrasound-guided rhomboid intercostal block (RIB) in alleviating postoperative pain has been well concerned. This study aims to compare the effectiveness between ultrasound-guided RIB and paravertebral block (PVB) in alleviating acute pain following video-assisted thoracic surgery.

**Methods:**

It was a prospective, randomized, double-blinded clinical trial involving 132 patients with video-assisted thoracic surgery divided into three groups: the general anesthesia (GA) group, RIB group, and PVB group on T5 vertebra, using 0.4% ropivacaine at 3 mg/kg, registered in the Chinese Clinical Trial Registry (ChiCTR2100054057, “https://www.chictr.org.cn”). The visual analogue scale (VAS) scores at rest and cough during 48 h postoperatively and the postoperative consumption of pain rescue were the primary outcomes, and the QoR15 score 48 h postoperatively, the usage of opioids during and after operation, and nerve block-related complications were the secondary outcomes. Demographic characteristics, surgery characteristics, and primary outcomes between the groups were compared.

**Results:**

A total of 120 eligible patients were recruited, including 40 in each group. Baseline and surgery characteristics between the groups were comparable (all *p* > 0.05). The PVB and RIB groups were better than the GA group in the primary and secondary outcomes (*p* < 0.05). The static VAS score, QoR15 score, and block-related complications within 48 hours after surgery were better in the RIB group than in the PVB group (*p* < 0.001).

**Conclusion:**

Both PVB and RIB can provide adequate analgesia and accelerate the recovery of patients. Compared with PVB, RIB has a better analgesic effect, especially to avoid paravertebral pain caused by block, and the operation of RIB is more straightforward and the safety is higher.

## 1. Introduction

Thoracic surgery is specialized in the study of the chest organs and mainly refers to the lung, esophagus, and mediastinum diseases diagnosis and treatment, such as chest trauma, lung tumors, esophageal tumors, chronic obstructive emphysema, tuberculosis, esophageal functional diseases, diaphragmatic diseases, and congenital diseases of the chest. So many patients need to be treated with thoracic surgery in the world. Compared to thoracotomy, video-assisted thoracic surgery (VATS) has similar therapeutic effects with less invasion and relieves postoperative pain significantly, promoting rehabilitation [1–3]. However, the VATS still causes moderate to severe postoperative pain [4, 5]. Postoperative analgesia is a critical issue in accelerating the rehabilitation of patients during the perioperative period. The thoracic epidural block (TEB) is the gold standard for analgesia for thoracic surgery. However, the complications such as hypotension and nerve injury caused by it have been a problem that cannot be ignored. The PVB is currently considered an optimal alternative to TEB because of the same analgesia effect and higher safety [6, 7]. But the PVB also comes with some complications, such as pneumothorax, intercostal vascular injury, and paraspinal muscle pain [8–11]. Ultrasound-guided serrate anterior plane block (SAPB) is another strategy for alleviating postoperative pain, however, is noninferior to the PVB in analgesic for both acute and chronic pain 1-year post thoracoscopic surgery, and the SPAB applied safer and more patient-satisfying pain relief regimens [12]. Compared to PVB, fascial blocks are safer and easier to be performed. Also, more and more fascial blocks have been widely used in thoracic analgesia because of their convenient operation and reliable analgesia. The ultrasound-guided rhomboid intercostal block (UG-RIB) is one of them, which was discovered by Elsharkawy et al. in 2016 [13]. It has been reported that UG-RIB can ensure adequate analgesia in modified radical mastectomy and VATS [11, 14, 15]. However, a comparison of the analgesic efficacy between RIB and PVB has yet to be reported. Therefore, it is urgent to compare the clinical effects of RIB and PVB in alleviating acute pain following video-assisted thoracic surgery to provide a scientific and practical theoretical foundation for selecting a subsequent analgesic strategy. It is of great significance to improve patients' life and rehabilitation and avoid other complications after the operation.

Hence, we conducted this double-blinded, randomized, controlled study to compare the analgesic efficacy of RIB with that of PVB in VATS as an analgesic supplement to general analgesia, taking the acute VAS score and postoperative consumption of pain rescue as the significant outcome, and the QoR15 score 48 h postoperatively, the usage of opioids during and after operation and nerve block-related, complications were the secondary outcomes. Demographic characteristics, surgery characteristics, and primary outcomes between the groups were compared. This study found that both PVB and RIB can provide adequate analgesia and accelerate the recovery of patients. Compared with PVB, RIB has a better analgesic effect and fewer complications, especially to avoid paravertebral pain caused by block.

## 2. Patients and Methods

### 2.1. Subjects

This study was approved by the Ethics Committee of Nanjing Drum Tower Hospital (No. 2018-160-02) and registered in the Chinese Clinical Trial Registry (ChiCTR2100054057, https://www.chictr.org.cn, June 5, 2022; Yan Wang, M D.). Clinical trial procedures followed the principles of the Declaration of Helsinki.

A total of 132 patients with lung nodules receiving VATS with general anesthesia (the GA Group), ultrasound-guided RIB (RIB Group), or PVB (the PVB Group) in the plane of the T5 level using 0.4% ropivacaine (Zhejiang Xianju Pharmaceutical Co., Ltd., Zhejiang, China) at 3 mg/kg between June 8th, 2022, and August 10th, 2022, were recruited.

Inclusion criteria are as follows: (1) 18–80 years of age; (2) American Society of Anesthesiologists (ASA) Class I-III; and (3) written informed consent was obtained.

Exclusion criteria are as follows: (1) Allergy to local anesthetics, nonsteroidal anti-inflammatory drugs, and opioids; (2) infection of the skin at the puncture site; (3) peptic ulcer disease or inflammatory bowel disease; (4) renal deficiency; (5) transferring to thoracotomy; (6) daily use of opioids; and (7) bilateral operation.

### 2.2. Blinding

Eligible patients were randomly assigned to the three groups: the general anesthesia (GA) group, the RIB group, and the PVB group. A statistician who was not involved in the data analysis prepared a computer-generated list of random numbers and sealed them in separate envelopes. The list of random numbers determines the allocation.

**Figure 1:**
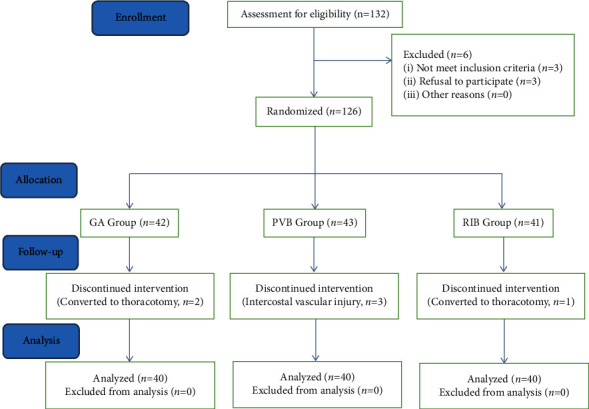
Flow diagram.

The study coordinators, attending anesthesiologists and postoperative follow-up personnel, and the patients were all blinded to the group assignment. Anesthesia induction and nerve blocks for all patients were performed by a group of independent experienced anesthesiologists in the preanesthesia room according to the random number before the operation (the GA group, only performed induction). Afterwards, the patients were transferred to the operating room to start the operation. In PACU, the block dermatome region is defined before the patients leave the operation room. The postoperative analgesia regimen comprises an infusion of flurbiprofen and patient-controlled intravenous sufentanil. Demographic data and surgical and anesthetic data were recorded. Postoperative VAS scores, consumption of opioids, and QoR15 scores were documented to estimate the analgesic effect. The recruitment of subjects is depicted in Figure 1.

### 2.3. Anesthetic and Surgical Management

#### 2.3.1. Anesthesia Introduction and Maintenance

Anesthesia introduction was performed by the administration of 2–3 mg/kg propofol (produced by Fresenius Kabi Austria GmbH, subpackage by Beijing Fresenius Capi Medical Co., Ltd., Beijing, China), 0.1 mg/kg vecuronium bromide (Yangzijiang Pharmaceutical Group Co., Ltd., Jiangsu, China), and 2–5 *μ*g/kg fentanyl (Hubei Renfu Pharmaceutical Group Co., Ltd., Hubei, China). After intubation, mechanical ventilation was supported at the end-tidal CO_2_ (ETCO2) of 35–40 mmHg and SpO_2_ of 95%–100% with 50–100% oxygen concentration. Anesthesia maintenance was performed by the administration of 2% propofol at 4–10 mg/kg/h titrated to the bispectrality index within 40–60, remifentanil (Hubei Renfu Pharmaceutical Group Co., Ltd., Hubei, China) at 0.1–0.4 *μ*g/kg/h, and cisatracurium (Jiangsu Hengrui Pharmaceutical Co., Ltd., Jiangsu, China) at 0.06–0.12 *μ*g/kg/h. Intravenous administration of fentanyl at 2–4 *μ*g/kg was intraoperatively given if necessary.

#### 2.3.2. Surgical Management

Patients in the three groups were scheduled for wedge resection, segment resection, and lobectomy, which were determined by preoperative chest CT and intraoperative pathological results. All operations were performed by the same group of surgeons. All surgical procedures were completed by video-assisted thoracic surgery (VATS) with two ports. The trochal ports were made at the fourth/seventh intercostal levels.

All the resection material was removed by a surgical glove without excessive expansion of the incision. A chest drain (22F) was inserted at the seventh intercostal level before the skin closure. When suturing the wound, carefully identify the position of the intercostal nerve to avoid injuring the intercostal nerve. No wound retractor was used during the operation.

#### 2.3.3. Ultrasound-Guided Nerve Blocks

Patients in the PVB and RIB groups were intervened by ultrasound-guided nerve block in the lateral position with local anesthesia. The PVB was performed using the in-plane technique with a linear 4–10 MHz ultrasound probe (LOGIQe, GE Healthcare, Waukesha, WI., U.S.A.). At the parasagittal view, subcutaneous tissues, T5 transverse processes, superior costotransverse ligament (SCTL), and pleura were visualized. An 18 G block needle was inserted vertically or slightly caudally into the paravertebral space (PVS) under the guidance of ultrasound. After the penetration of the SCTL, a slight aspiration was performed to ensure the avoidance of vessels or pleura. Then, 1–2 ml of normal saline was injected into the PVS, the pressure of which pushed down the pleura. The position of the needle tip was confirmed, and 0.4% ropivacaine (Zhejiang Xianju Pharmaceutical Co., Ltd., Zhejiang, China) at 3 mg/kg was injected into the PVS.

A linear 4–10 MHz ultrasound probe (LOGIQe, GE Healthcare, Waukesha, WI, U.S.A.) was placed on the medial border of the scapula between the 4th and 5th rib of the patients in the RIB group. In the ultrasound image, the trapezius muscle, rhomboid muscle, intercostal muscles, pleura, and lung were identified. Under the aseptic condition, an 18 G block needle was inserted laterally in the plane of the T5 level guided by an ultrasound probe with an in-plane technique. The vessel injection should be confirmed negative through aspiration, and 1–3 ml of normal saline was injected to divide the rhomboid and intercostal muscle, and 0.4% ropivacaine at 3 mg/kg was injected into the deep layer of the rhomboid muscle.

#### 2.3.4. Pain Management

At the end of the operation, 50 mg of flurbiprofen axetil injection (Beijing Taide Pharmaceutical Co., Ltd., Beijing, China) was intravenously administrated. On the 1st and 2nd day postoperatively, the flurbiprofen axetil injection was given intravenously with doses of 50 mg Bid. Besides intravenous infusion of flurbiprofen axetil injection, postoperative analgesic protocol composes of intravenous patient-controlled sufentanil analgesia rescue, setting a bolus dose at 2 *μ*g/2 mL (1 *μ*g/mL, total volume 100 mL) (Hubei Renfu Pharmaceutical Group Co., Ltd., Hubei, China), with a lock time of 30 min.

### 2.4. Outcome Measures

The primary outcomes were the visual analogue scale (VAS) scores at rest and cough during 48 h postoperatively and the postoperative consumption of pain rescue. The secondary outcomes include the QoR15 score at 24 h and 48 h postoperatively, data related to the usage of opioids during and after an operation, and nerve block-related complications, such as hypotension, vascular injury, and muscle pain at the injection site. Intraoperative hypotension was defined as a decrease in mean arterial pressure greater than 20% of the baseline. Additionally, the dermatomal distribution of the sensory blockade (area between anterior axillary and middle axillary) was collected.

### 2.5. Sample Size

This pilot study has a sample size of 25 patients per group. The average consumption of sufentanil for analgesia within 24 hours after surgery in the three groups was 62.3 ± 7.8 *μ*g, 51.5 ± 6.8 *μ*g, and 48.8 ± 5.3 *μ*g. Assuming an alpha error of 0.01 (two-tailed) and a power of 0.90, a minimum of 27 participants per group was calculated using PASS software. A 20% follow-up failure rate was expanded to include 33 samples in each group. During the implementation of the experiment, the number of eligible patients in each group reached 40, and the final sample size in each group was determined to be 40.

### 2.6. Statistical Analysis

Data were analyzed using SPSS version 25.0 (IBM Corp., Armonk, NY, USA). Continuous data were inspected and tested for distribution using the Shapiro–Wilk test. Normally distributed data were analyzed by ANOVA to compare the three groups (the GA group, the PVB group, and the RIB group) with differences in the outcome parameters. Normally distributed data are presented as mean ± standard deviation. Numerical data of the two groups were compared using the Student's *t*-test or the Mann–Whitney test, depending on whether the data were distributed normally or not. Chip-square test or Wilcoxon rank-sum test loaded inside SPSS 22.0 was used for statistical processing, and *p* < 0.05 was considered as statistically significant.

## 3. Results

During the whole process of a clinical trial, three patients in the PVB group suffered intercostal artery bleeding due to a PVB puncture. Considering the possibility of local anesthetic absorbed by the blood, we were forced to give up the paravertebral block and excluded these three patients. There were two cases in the GA group, and one case in the RIB group was converted to thoracotomy, which was also excluded from the statistical cases. Finally, a total of 120 consenting patients enrolled completed all the perioperative assessments (Figure 1). They were randomly assigned to the three groups, with 40 in each.

### 3.1. Demographic Characteristics and Surgery Characteristics between the Three Groups

Preoperative baseline characteristics of the patients between the three groups were recorded and compared. As shown in Table 1, the age, height, body weight, sex, and ASA class were comparable between the three groups (the *p* value range from 0.3170 to 0.9884, all *p* > 0.05).

We next analyzed surgery characteristics between the three groups. No significant differences in the types and surgical direction of operation, surgery time, drainage time of chest tube, and postoperative length of stay were detected between the three groups (the *p* value range from 0.2184 to 0.9663, all *p* > 0.05, Table 1).

### 3.2. Postoperative VAS Score, the Dosage of Perioperative Opioids, and the QoR15 Score

**Figure 2:**
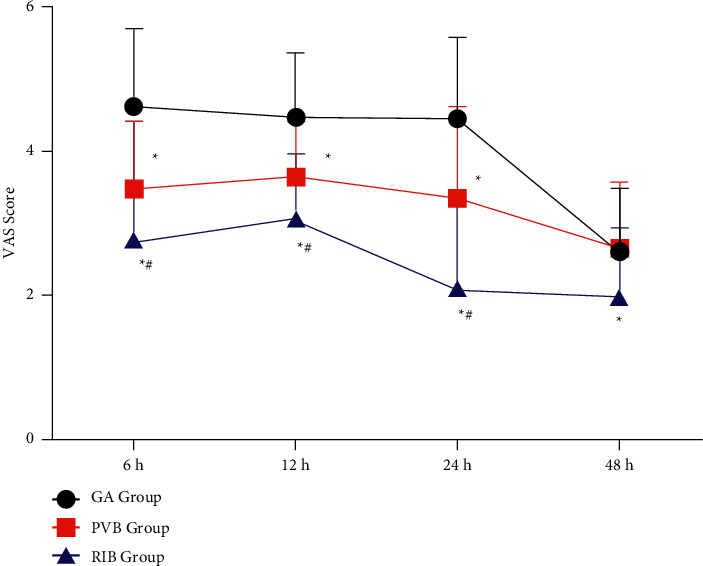
VAS score at rest post operation. GA, general anesthesia; PVB, thoracic paravertebral; RIB, rhomboid intercostal block; ^*∗*^*p* < 0.05 when compared with the GA group, ^#^*p* < 0.05 when compared with the PVB group.

**Figure 3:**
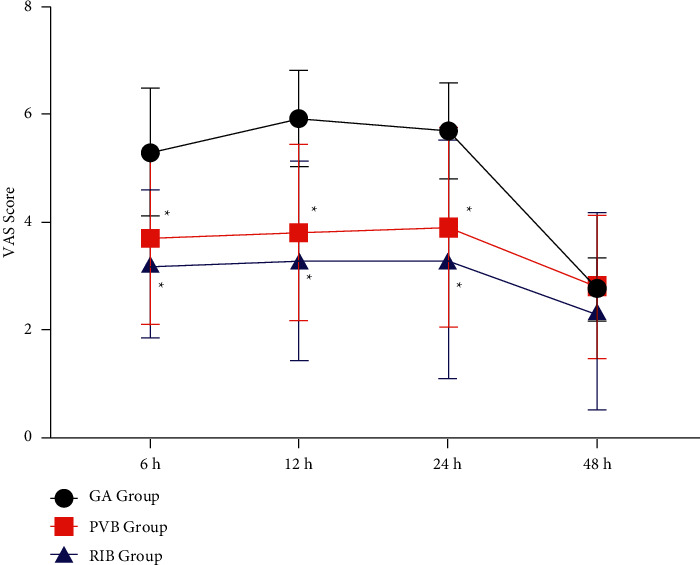
VAS score on movement postoperation. GA, general anesthesia; PVB, thoracic paravertebral; RIB, rhomboid intercostal block; ^*∗*^*p* < 0.05 when compared with the GA group.

The VAS scores at rest at 6 h, 12 h, and 24 h after operation in the GA group were significantly higher than those in the PVB and RIB groups (*p* < 0.001, Figure 2). The VAS score at the rest of the PVB group was significantly higher than that of the RIB group at 6 h, 12 h, and 24 h after operation (*p* < 0.001, Figure 2). At 48 hours after the operation, the VAS score at rest in the RIB group was significantly lower than that in the GA group and the PVB group (*p* < 0.001, Figure 2), and there was no significant difference between the GA group and the PVB group (*p*=0.078, Figure 2). At 6 h, 12 h, and 24 h after the operation, the VAS score on cough in the GA group was significantly higher than that in the PVB group and the RIB group (*p*=0.032, Figure 3). There was no significant difference between the PVB and RIB groups (*p*=0.088, Figure 3). 48 hours after the operation, there was no statistically significant difference in the VAS score on cough between the three groups (*p*=0.083, Figure 3).

The intraoperative fentanyl dosage in the GA group was significantly higher than that in the PVB group and the RIB group (*p* < 0.001, Table 2). There was no significant difference between the PVB and RIB groups (*p* > 0.05, Table 2). There was no significant difference in intraoperative remifentanil dosage among the three groups (the GA group vs. the PVB group *p*=0.6606; the GA group vs. the RIB group *p*=0.4591; the PVB group vs. the RIB group *p*=0.9436; Table 2).

The onset time of sufentanil for rescue analgesia was significantly earlier in the GA group than in the PVB and RIB groups (*p* < 0.001, Table 2). There was no significant difference between the PVB and RIB groups (*p*=0.6900, Table 2).

**Figure 4:**
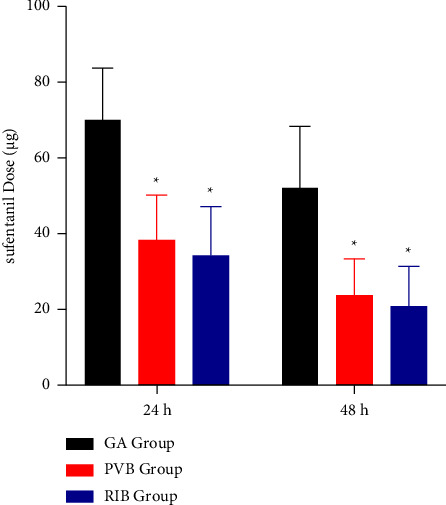
Sufentanil dose for pain rescue post operation. GA, general anesthesia; PVB, thoracic paravertebral; RIB, rhomboid intercostal block; ^*∗*^*p* < 0.05 when compared with the GA group.

At 24 hours and 48 hours after the operation, the dose of sufentanil for rescue analgesia in the GA group was significantly higher than that in the PVB group and the RIB group (*p*=0.031, Figure 4). There was no significant difference between the PVB and RIB groups (*p*=0.2175, Figure 4).

The QoR15 scores at 24 hours and 48 hours after operation in the GA group were significantly lower than that in the PVB group and the RIB group (*p* < 0.001, Table 2). In the PVB group, the QoR15 scores at 24 hours and 48 hours postoperation were significantly lower than those in the RIB group (*p* < 0.001, Table 2).

### 3.3. Data Related to Nerve Blocks

The incidence of hypotension accompanied by the nerve block is lower in the RIB group than that in the PVB group (*p*=0.037, Table 3).

The incidence of vascular damage and muscle pain at the injection site related to the nerve block is significantly lower in the RIB group than that in the PVB group (*p*=0.028, Table 3).

Although there was no statistically significant difference in the block range between the PVB group and the RIB group (*p*=0.1893, Table 3), three patients in the PVB group had the block area not covering the thoracic drainage tube wound, resulting in significant postoperative pain in the drainage tube wound.

## 4. Discussion

The gold standard of traditional thoracic analgesia is the epidural block (TEB). Compared with the epidural block, the PVB has been found to have the same analgesic effect and fewer complications, and so it is regarded as the best alternative to the epidural block. It has also become the object of comparison of the effectiveness of the most new thoracic nerve block techniques. Most of the previous studies [11, 13–15] on RIB are to observe the analgesic effect of patients before and after the implementation of RIB or the comparison between RIB and other fascial blocks (the erector spinae plane block and the serratus plane block). These studies show that RIB can be used for corresponding surgical analgesia, but there are few comparative studies between RIB and standard thoracic analgesia, such as PVB. This prospective double-blinded randomized controlled trial has compared the GA, PVB, and RIB groups in the dynamic and static pain scores and QoR15 within 48 hours, as well as the intraoperative fentanyl and postoperative sufentanil consumption, and complications with PVB/RIB found that compared with the GA group, the PVB and RIB groups consume fewer opioid drugs for analgesia and have more advantages in the VAS score and the QoR15 score, which indicates that both types of block can promote patients' rehabilitation that the RIB has a better analgesic effect, especially to avoid paravertebral pain caused by block. Compared with the PVB group, the RIB has a better analgesic effect and fewer complications and can be a good substitute for PVB in some cases where PVB is contraindicated.

Although there was no significant difference in the dynamic pain score within 48 hours between the PVB and RIB groups, the static pain score in the PVB group was higher than that in the rib group, which was mainly attributed to the paravertebral muscle pain at the injection site in some patients (7/40) in the PVB group. One of the patients still complained of muscle pain at the position of the paravertebral block at the follow-up one month after the operation. This is because the position of the paravertebral space is deeper than the space between the rhomboid muscle and the intercostal space, and the nerve block needle needs to cross more layers of the tissue to reach the block position, resulting in more severe tissue damage, especially in repeated cases, the paravertebral muscle pain would worsen. Similar clinical results have also been reported in the previous studies [11, 16, 17].

Clinical studies [18–20] have confirmed that the adjacent intercostal paravertebral spaces communicate with each other, which provides an anatomical basis for spreading a local anesthetic to the adjacent intercostal space. Saito et al. noted that an average single-shot injection volume of 2 mL is required to provide unilateral PVB for each dermatome level [21]. However, Cheema et al. have expounded on the unpredictable dermatomal spread of a single-shot paravertebral blockade injection [22]. Not any single-point block can produce local anesthetic spread longitudinally along the vertebral column to block multiple intercostals [23]. Local anesthetic drugs may be limited to a single intercostal or coexist with diffusion along the long axis of the spine or even diffuse into the spinal canal. From our observations, although there was no statistical difference in the coverage of the intercostal segment between the PVB and RIB groups, three patients in the PVB group had a chest tube wound that was not completely blocked. In contrast, all patients in the RIB group were effectively blocked in the intercostal space of the drainage tube wound. We consider that it relates to the uncertainty of the diffusion mode of PVB after the injection of local anesthetics. In contrast, the RIB can easily obtain enough range of intercostal blocks. Local anesthetic drugs with sufficient capacity can spread across multiple intercostals between the two layers of muscle in a single injection.

In addition, the probability of intraoperative hypotension in the PVB group was also higher than that in the RIB group. We also believe this is related to the diffusion mode of local anesthetic drugs in the PVB. Although the PVB transfers the block site from the intraspinal to the paravertebral triangle compared with TEB, local anesthetic drugs could diffuse into the spinal canal from the paravertebral triangle. Local anesthetics into the spinal canal may block the sympathetic nerve and cause blood pressure to drop [7]. The injection site of the RIB is far away from the paravertebral triangle, the local anesthetics will not diffuse into the spinal canal, and the incidence of RIB-related hypotension during operation is very low.

In this experiment, there were 3 cases in which paravertebral vascular injury occurred in the paravertebral block under ultrasound, and the local anesthetic could not continue to be administered. Song et al. reported a similar case of PVB with severe intercostal vascular injury [8]. Many clinical studies confirmed that the paravertebral triangle area where PVB is performed has a high incidence of anatomical variation in the intercostal arteries [24–29]. Also, there are still various clinical situations that make it challenging to find the course of intercostal blood vessels under ultrasound [30]. Two factors will lead to accidental iatrogenic intercostal blood vessel injury. In contrast, the block site of the RIB block rarely involves vascular variations, and the safety is relatively high.

Postoperative analgesia is a good starting point to accelerate postoperative rehabilitation. From the results of this study, it is concluded that the postoperative pain score in the RIB group and the PVB group is lower than that in the GA group. Furthermore, the incidence of postoperative paraspinal muscle pain in the RIB group is lower than that in the PVB group; the postoperative pain score is lower. Compared with simple intravenous drug analgesia, the nerve block reduces the dosage of opioid drugs on the basis of ensuring better analgesia that helps patients to go to the ground as soon as possible, promotes the recovery of gastrointestinal function, and avoids the occurrence of deep vein thrombosis and quickly return to the state of life before the operation. Correspondingly, the QoR15 score covers all essential factors of rapid rehabilitation and has been widely used in postoperative recovery evaluation. In this study, the QoR15 score of the PVB group and the RIB group was higher than that of the GA group within 48 hours after the operation, while that of the RIB group was higher than that of the PVB group. This shows that both PVB and RIB can promote the postoperative rehabilitation of patients primarily since the RIB provides adequate analgesia and has fewer block-related complications. The QoR15 score post operation is higher than that of the other two groups.

The different anatomical basis of the PVB and RIB lead to other block characteristics from the two blocks. Compared with the TEB, the PVB is the transfer of blocks from the intraspinal to the extravertebral triangle through the skin, subcutaneous tissue, paraspinal muscle, and costal transverse process ligament and finally reach the paraspinal triangle to inject a local anesthetic into it. The RIB injects local anesthetic into the space between the rhomboid muscle and the intercostal muscle in the area between the spine and the scapula. The block site is shallower than PVB, and the tissue damage is slighter than PVB; the incidence of paraspinal muscle pain is extremely low. Because the paravertebral triangle is adjacent to the intervertebral foramen, there are also intercostal arteries and their branches with significant variation, intercostal vascular injury, and local anesthetic drugs enter the spinal canal and cause hypotension are common complications of PVB. The block site of the RIB is far away from the paravertebral triangle to avoid the abovementioned complications. As a kind of fascial block, the RIB can obtain the range of multiple intercostal blocks with a local anesthetic drug spreading multiple intercostal segments easily between the two layers of muscle in a single intercostal block. In comparison, the range of blocks caused by a single intercostal block of PVB has intense uncertainty.

In conclusion, compared with the simple general anesthesia group, both PVB and RIB can reduce perioperative opioid consumption, provide adequate analgesia for patients, and accelerate their recovery of patients. Through this double-blinded, randomized, controlled study, we have first compared the efficacy between standard thoracic analgesia (such as PVB) and ultrasound-guided RIB and found that RIB has a better analgesic effect, especially to avoid paravertebral pain caused by block, and the operation of RIB is more straightforward and the safety is higher. Through this study, we can provide guidelines for analgesic management of acute pain after video-assisted thoracic surgery.

## Figures and Tables

**Table 1:** Demographic characteristics and surgery characteristics between three groups (*n* = 120).

	GA group (*n* = 40)	PVB group (*n* = 40)	RIB group (*n* = 40)	*p* value
Age (year)	60.775 ± 12.160	61.125 ± 8.504	57.625 ± 11.293	>0.05
Height (m)	1.645 ± 0.087	1.656 ± 0.064	1.643 ± 0.063	>0.05
Body weight (kg)	66.025 ± 10.614	64.175 ± 8.924	66.150 ± 12.052	>0.05
Male (*n*, %)	20 (50%)	20 (50%)	20 (50%)	>0.05
ASA (II/III)	20/20	21/19	21/19	>0.05
Hypertension (*n*, %)	17 (42.5%)	22 (55%)	15 (37.5%)	>0.05
CAD (*n*, %)	3 (7.5%)	1 (2.5%)	2 (5%)	>0.05
Diabetes	4 (10%)	3 (7.5%)	6 (15%)	>0.05
Duration of surgery (min)	103.700 ± 35.930	103.700 ± 35.930	103.700 ± 35.930	>0.05
Right side surgery (*n*, %)	18 (45%)	18 (45%)	19 (47.5%)	>0.05
Surgical procedure				
Wedge resection	8 (20%)	11 (27.5%)	9 (22.5%)	>0.05
Segment	9 (22.5%)	9 (22.5%)	7 (17.5%)	
Lobectomy	17 (42.5%)	15 (37.5%)	17 (42.5%)	
Mediastinal mass resection	6 (15%)	5 (12.5%)	7 (17.5%)	
Chest tube drainage (hrs)	46.625 ± 5.646	44.975 ± 4.699	44.088 ± 4.904	>0.05
Hospital stay (days)	3.225 ± 0.660	3.003 ± 0.622	3.050 ± 0.541	>0.05

GA, general anesthesia; PVB, thoracic paravertebral; RIB, rhomboid intercostal block; ASA, American Society of Anesthesiologists; CAD, coronary heart disease.

**Table 2:** Perioperative opioid consumption and Qo15 score between the three groups (*n* = 120).

	GA group (*n* = 40)	PVB group (*n* = 40)	RIB group (*n* = 40)	*p* value
Fentanyl dose (mg)	0.598 ± 0.169	0.394 ± 0.113	0.405 ± 0.132	<0.001
Remifentanil dose (mg)	0.765 ± 0.208	0.690 ± 0.450	0.662 ± 0.448	>0.05
Time to first pain rescue (h)	2.457 ± 0.781	11.930 ± 1.241	12.605 ± 2.301	<0.001
QoR15 24 h	86.457 ± 5.840	94.600 ± 6.764	111.800 ± 7.959	<0.001
QoR15 48 h	110.475 ± 4.941	115.400 ± 6.747	130.200 ± 7.251	<0.001

GA, general anesthesia; PVB, thoracic paravertebral; RIB, rhomboid intercostal block; QoR, quality of recovery.

**Table 3:** Data related to PVB and RIB (*n* = 80).

	PVB group (*n* = 40)	RIB group (*n* = 40)	*p* value
Hypotension (*n*, %)	8 (20%)	2 (5%)	<0.05
Vascular damage (*n*)	3(43)^*∗*^	0 (40)	<0.001
Muscle pain at the injection site (*n*, %)	7 (17.5%)	0 (0%)	<0.001
Dermatomal block distribution	3.400 ± 0.778	5.150 ± 0.949	>0.05
Missing the intercostal space where drainage tube located (*n*, %)	3 (7.5%)	0 (0%)	<0.001

PVB, thoracic paravertebral; RIB, rhomboid intercostal block; ^*∗*^Two patients in the PVB group developed vascular injury and were unable to continue bolus injection of local anesthesia.

## Data Availability

The data used to support the findings of this study are available from the corresponding author upon request.

## Supplementary Materials

Supplementary Materials The raw data of the manuscript are in the supplementary file.
